# Non-Pharmaceutical Interventions on COVID-19 in Workers and Residents of Nursing Homes in Geneva: A Mixed Qualitative and Quantitative Study

**DOI:** 10.3390/epidemiologia6010014

**Published:** 2025-03-11

**Authors:** Lakshmi Krishna Menon, Ania Wisniak, Simon Regard, Silvia Stringhini, Idris Guessous, Jean-François Balavoine, Omar Kherad, The SEROCoV-WORK + Study Group

**Affiliations:** 1 Unit of Population Epidemiology, Division of Primary Care Medicine, Geneva University Hospitals, 1205 Geneva, Switzerland; 2 Institute of Global Health, Faculty of Medicine, University of Geneva, 1205 Geneva, Switzerland; 3 Department of Security, Population and Health, General Health Directorate, Canton of Geneva, 1211 Geneva, Switzerland; simon.regard@hcuge.ch; 4 Division of Emergency Medicine, Geneva University Hospitals, 1205 Geneva, Switzerland; 5 Department of Health and Community Medicine, Faculty of Medicine, University of Geneva, 1205 Geneva, Switzerland; 6 University Center for General Medicine and Public Health, University of Lausanne, 1015 Lausanne, Switzerland; 7 School of Population and Public Health and Edwin S.H. Leong Centre for Healthy Aging, Faculty of Medicine, University of British Columbia, Vancouver, BC V6T 1Z4, Canada; 8 Division of Primary Care Medicine, Geneva University Hospitals, 1205 Geneva, Switzerland; 9 Department of Medicine, Faculty of Medicine, University of Geneva, 1205 Geneva, Switzerland; 10 Division of Internal Medicine, Hôpital de la Tour, 1217 Meyrin, Switzerland

**Keywords:** non-pharmaceutical interventions, COVID-19, nursing homes, long-term care, Geneva, infection control

## Abstract

The objective of this study was to examine the impact of varying levels of non-pharmaceutical interventions (NPIs) on COVID-19 transmission in nursing homes during the first wave of the pandemic. Background/Objectives: The primary aim involved exploring qualitative insights from staff and management regarding the implementation of NPIs. The secondary aim was to determine the cumulative incidence of PCR-confirmed COVID-19 cases among residents. Incident rate ratios (IRRs) were the calculated levels of NPI restrictiveness. Methods: We used a mixed methodology to identify factors that might have affected COVID-19 expansion in nursing homes in the canton of Geneva, Switzerland. For the qualitative component, we interviewed the Attending Physicians and/or Director of each nursing home. In the quantitative component, we calculated incident rate ratios (IRRs) for infection between the three levels of COVID-19-related measures taken in these nursing homes, and the cumulative incidence of PCR-confirmed COVID-19 cases in their resident population. This study was conducted in 12 nursing homes located in the canton of Geneva, Switzerland, between 1 March 2020, and 1 June 2020. Results: Most nursing homes mandated NPIs for their staff and residents during the first wave of COVID-19. We found an equal distribution of maximally (*n* = 4), moderately (*n* = 4), and minimally (*n* = 4) restrictive NPIs for nursing home workers and residents. The extent of NPIs implemented was not shown to be significantly associated with the cumulative incidence of COVID-19 cases among residents (maximally restrictive IRR = 3.90, 95%CI 0.82–45.54, *p* = 0.184; moderately restrictive IRR = 3.55, 95%CI 0.75–41.42, *p* = 0.212; minimally restrictive IRR = reference). Conclusions: Nursing homes in our study showed high variability in which NPIs, and to what extent, they implemented, with no significant relationship between the restrictiveness of NPIs and COVID-19 incidence among nursing home residents. This suggests that other factors influence the transmission of COVID-19 in these settings. Future research should explore additional determinants and the balance between strict NPIs and the overall well-being of residents.

## 1. Introduction

At the beginning of the COVID-19 pandemic, nursing homes were disproportionately affected due to their shared living spaces and the heightened vulnerability of their residents. Many of these residents, typically older adults with pre-existing chronic health conditions, were especially at risk for severe COVID-19 outcomes, such as hospitalization and death [1]. Contributing factors included the widespread prevalence of these underlying conditions, insufficient access to personal protective equipment, and difficulties in implementing effective infection control measures, all of which played a significant role in the swift transmission of the virus within these facilities. In Switzerland, nursing homes accounted for half of all deaths linked to COVID-19 [2]. A previous SEROCoV-WORK study, which examined seropositivity rates among essential workers in Geneva during the first wave of the pandemic, found a strong positive correlation between the percentage of seropositive staff in each nursing home and the cumulative incidence of COVID-19 cases, hospitalizations, and deaths among residents [2,3]. Also, the seroprevalence rates in nursing home staff were approximately 90-fold higher than the reported number of COVID-19 cases in nursing home staff, suggesting that many SARS-CoV-2 transmission cases occur between asymptomatic staff and residents within nursing homes [2]. This paper offers a qualitative narrative to situate these findings within real-world long-term care settings.

There are still scarce systematic data to understand disparities in COVID-19 cases and the risk factors for infection spread in nursing homes. Previous North-America-based studies have found that chronic disease and related atypical presentation of symptoms, as well as contact with asymptomatic direct-care workers, make nursing home residents particularly vulnerable to infection [4]. Other predictors are facility-specific, such as staffing hours, resident-to-staff ratio and the implementation of non-pharmaceutical interventions (NPIs) [5,6,7]. NPIs were at the forefront of outbreak control when vaccines and/or treatments were not available. Examples of NPIs include type and frequency of testing for infection, use of personal protective equipment (PPE), physical distancing, and other national strategies. An epidemiological model of the effect of major non-pharmaceutical interventions across 11 European countries, including Switzerland, found that physical distancing and national lockdowns have had an effect in decreasing COVID-19 transmission [6]. However, since most NPIs were implemented in tandem or in rapid succession with no regulation, and are largely a function of human adherence, it remains difficult to disentangle the extent of both macro- and micro-effect sizes of each intervention.

The objective of this mixed-methodology study was to assess precisely which NPIs, and to what extent, individual nursing homes followed for their staff and residents during the first wave of COVID-19 in the canton of Geneva, Switzerland. There was also an imminent need to understand how these NPIs were perceived and implemented during the first wave of COVID-19, and whether they were effective in the nursing home settings.

## 2. Materials and Methods

Nursing homes were included in this study if they were located in the Canton of Geneva and had healthcare workers enrolled in the aforementioned SEROCoV+ WORK study [3]. Nursing homes were excluded from this study if they withdrew consent to participate in either, or both, the interview or the demographic survey. Out of the 25 nursing homes eligible, our final study population consisted of 12 institutions that agreed to participate. We interviewed the Attending Physicians and/or Director of these nursing homes in Geneva to assess COVID-19 Screening Strategies, Nursing Home Policies/Management, and COVID-19 Infection Control Measures to identify factors that might have affected COVID-19 expansion in their nursing homes during the first wave between 1 March 2020 and 1 June 2020 (Appendix A). Psychometric properties of the semi-structured questionnaires were assessed by consulting a nursing home Director, who provided feedback on what overarching themes they thought the questionnaire intended to measure (Appendix B).

Formal consent of the attending Physician and Director was obtained prior to the semi-structured interview. The nursing homes were assured that this study was only exploratory of best anti-COVID-19 practices, not a critique of the approaches individual nursing homes used. All person-specific or institution-specific data collected were encoded.

The interviews were conducted in French, through videoconference or in person, between 24 March 2021 and 6 May 2021. Each interview lasted between 30 and 40 min. The questions retrospectively explored (1) COVID-19 Screening Strategies, (2) Nursing Home Policies/Management, and (3) COVID-19 Infection Control Measures, in each nursing home during the first wave of COVID-19, between 1 March 2020 and 1 June 2020 (Appendix A). Data collection leveraged the clinical expertise of this study’s principal investigator, who is a doctor in Geneva and the primary interviewer. The first author’s background in public health in Geneva influenced both the framing of the research questions and the interpretation of the findings. Nursing homes were also given a demographic survey to complete during, or after, the interview (Appendix B). This questionnaire collected baseline demographic data, including size of workforce, distribution of residents by age and/or sex, and number of personnel employed. The questionnaire and demographic survey can be found in the Appendix A and Appendix B.

Conversations were recorded and transcribed for text mining and thematic analyses guided by Braun and Clarke’s (2006) six-step framework for qualitative research [7]. We exclusively used in vivo coding, where the code itself is something the participant has said. In vivo coding also supports the dependability of our results as these codes are direct extractions from the interviews. This approach, termed a ‘inductive analysis’, was particularly relevant for this study because both interviewers and interviewees used phrases specific to NPIs in the nursing home context. Coding was performed manually to give full and equal attention to each non-pharmaceutical intervention, and candidate themes were refined in a re-iterative process with the research team. Using this qualitative case study approach under a constructivist paradigm is appropriate because it allows researchers to capture the complexities and nuances in how different nursing homes interpret, implement, and adapt NPIs. This acknowledges the diversity in experiences across settings and aims to build an understanding based on these varied perspectives, which is especially relevant when dealing with subjective, context-dependent data. Using both semi-structured interviews and demographic surveys provides a form of triangulation, enhancing reliability and credibility by combining multiple data sources.

**Figure 1:**
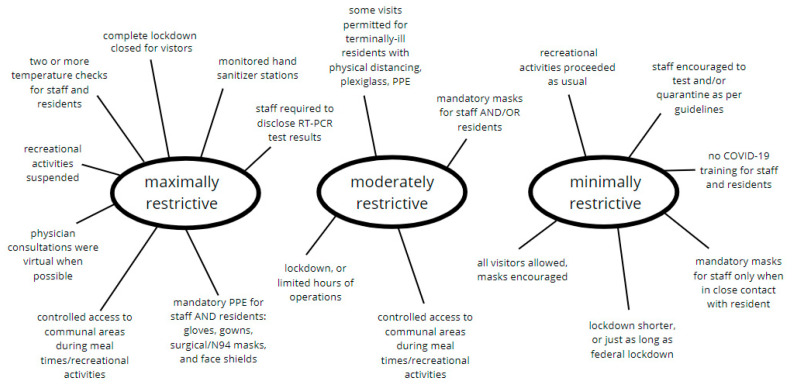
Thematic distribution of in vivo codes. Non-pharmaceutical interventions in each nursing homes were classified into three themes: maximally restrictive, moderately restrictive, or minimally restrictive.

In vivo codes formed an analytic narrative that represented three themes: maximally restrictive, moderately restrictive, and minimally restrictive. The classification of NPIs underwent a rapid validation process through face validity assessment by two study authors (L.K.M. and O.K.), both of whom have high expertise in public health and clinical practice. This ensured that the classification was systematically applied across all nursing homes despite variations in implementation. Non-pharmaceutical interventions in nursing homes were classified as maximally restrictive if they involved a combination of the following: complete lockdown, multiple daily screening strategies (temperature check, antigen or RT-PCR test, mandatory PPE, hand hygiene), restricted access to communal areas, and/or closed for visitations. Moderately restrictive measures included limited hours of operations, some daily screening strategies (temperature check, obligatory mask, hand hygiene), and controlled access to communal areas. Nursing homes that generally allowed their residents and staff to autonomously adopt anti-COVID-19 measures were classified under the ‘minimally restrictive’ thematic umbrella. Figure 1 displays the final thematic map created for this data set.

We also compared the thematic classification of nursing homes with maximally, moderately, and minimally restrictive NPIs with the cumulative incidence of PCR-confirmed COVID-19 cases in the resident population. Data on PCR-confirmed COVID-19 cases among residents of nursing homes in the canton of Geneva were obtained from the Department of Security, Population and Health of the canton of Geneva for the period between March and June 2020. This methodology was previously described in detail [2]. The association between the degree of NPIs (maximally, moderately or minimally restrictive) and the number of COVID-19 cases in the twelve nursing homes was assessed using quasi-Poisson log-linear regression models. The results of the regression models are presented as incidence rate ratios (IRRs) with their 95% confidence intervals. *p*-values lower than 0.05 were considered statistically significant. Statistical analysis was conducted using R statistical software (v. 4.0.3, R Foundation for Statistical Computing, Vienna, Austria).

## 3. Results

**Figure 2:**
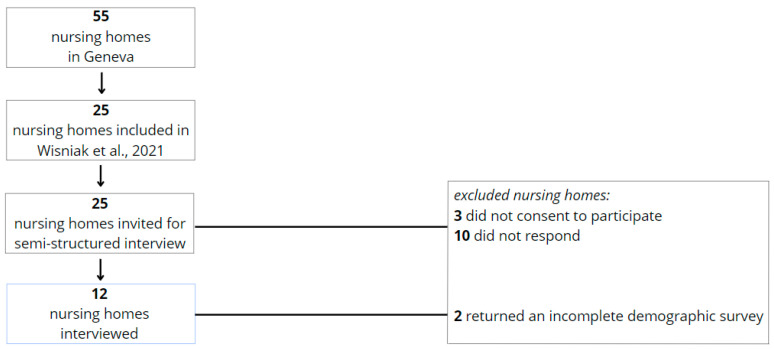
Flowchart depicting the inclusion and exclusion criteria applied to nursing homes, outlining the selection process for the final study population [2].

Of the 55 established nursing homes in Geneva, 25 were included in the previous SEROCoV-WORK study and had staff seroprevalence data available [3]. Of these, we interviewed 12, and analyzed the demographics of 10 (Figure 2).

### 3.1. Screening Strategies for Staff, Residents, and Visitors

Five nursing homes stated that they had no screening methods for their nursing home workforce. The same nursing homes also stated that they had no screening methods for their residents. Other screening strategies for staff included temperature checks at the reception, obligatory mask-wearing, and hand hygiene upon entry into the facility. Three interviewees mentioned specifically that though their workforce was encouraged to self-screen, and those with obvious COVID-19-compatible symptoms were referred to a general physician. It is unknown how often, and to what extent, these referrals were followed through.

Screening for residents more commonly involved between one and two temperature checks each day. Two nursing homes mentioned increased awareness to COVID-19 symptoms among residents, such as “fever, digestive issues, and cough”. While one nursing home had a dedicated unit for residents that tested positive for COVID-19, all other institutions asked residents to remain in their respective rooms if symptomatic.

Eight nursing homes remained under a lockdown during the first wave of COVID-19 in Geneva, each of which lasted a minimum of three weeks. However, these lockdowns varied in the spectrum of maximally restrictive to not restrictive. One interviewee stated that their nursing home started a lockdown one week in advance of the federal lockdown. Another home started welcoming visitors towards the end of April; meetings happened exclusively in their cafeteria, through a Plexi-glass shield. A third nursing home mentioned that they did not enforce a lockdown and allowed visitors, particularly for terminally ill residents.

### 3.2. Management of Staff and Residents Who Test Positive and/or Presented Symptoms Compatible with COVID-19

Ten nursing homes asked their workforce to quarantine at home for at least ten days if they tested positive for COVID-19 or came into contact with someone who tested positive. Two interviewees mentioned their workforce simply maintained a distance from residents if non-symptomatic.

Since most nursing homes did not have a dedicated COVID-19 unit, the residents remained isolated in their rooms until the symptoms subsided and/or an RT-PCR test returned negative. Two interviewees added that they restricted contact for symptomatic residents to two staff members, and another noted that their institution had a small team of COVID-19-trained staff. Four nursing homes admitted that none of their residents tested positive for COVID-19.

### 3.3. Shortages of Personal Protective Equipment

Eight nursing homes did not face any immediate shortages of personal protective equipment due to pre-existing stocks, though two interviewees admitted they were concerned about mask supplies. Four nursing homes added that they limited one mask and one gown per staff member per day. This meant their workforce was wearing single-use personal protective equipment for up to twelve hours a day. These shortages lasted between two months and fifteen days. During this time, one interviewee noted that their staff members only wore a mask when in direct contact with a resident.

### 3.4. Existing Infection Containment Policies

Five nursing homes did not have an existing outbreak protection plan before the COVID-19 pandemic. Other institutions relied on previous influenza outbreak plans, or general communications from cantonal health organizations. Most interviewees agreed that previous case management policies for outbreaks were effective during the first wave of the pandemic as these were already familiar to their workforce. One nursing home stated that they complied instead with a business continuity plan and a new organizational model to avoid staff absenteeism when switching to twelve-hour schedules.

### 3.5. Frequency of Social Interactions

Four homes reported they restricted all access to communal spaces during the first wave of COVID-19; meals were eaten in resident rooms directly, and visitations and all in-person activities were suspended. Four other interviewees controlled access to their communal spaces; residents had one socially distanced meal a day in the cafeteria, visitations took place in a dedicated space with obligatory mask-wearing, and in-person activities were limited to groups of five residents. The final four nursing homes reported all dining and recreation spaces remained open, and visitors were welcome with masks and appropriate hand hygiene.

The nursing homes did not require their residents to wear masks. Instead, the residents were encouraged to maintain distances of at least one meter, and sanitize their hands regularly. The interviewees reasoned that they were faced with making decisions that demanded a “compromise between health security and quality of life of residents for long-term health outcomes” during the first wave of COVID-19.

### 3.6. Nursing Home Demographics

Ten nursing homes provided baseline demographic statistics. Between March and May 2020, these institutions had an average of 74 staff present on site, including nurses, qualified and non-qualified caregivers, social workers, restaurateurs, housekeeping, therapists, volunteers, and administrative employees. Between 60 and 80% of these employees were female, and more commonly between 30 and 50 years of age. The percent of healthcare staff in these nursing homes that worked part-time hours ranged from 55% to 98%.

### 3.7. COVID-19 Information Dissemination

Five nursing homes arranged formal COVID-19 training and/or information sessions for their staff. Some of these workshops were led by medical personnel and covered best practices for infection control, including correct use of personal protective equipment. The same nursing homes also arranged informal discussions for thirty minutes with their residents, while another three institutions opted to distribute COVID-19 factsheets to residents and their families. Generally, “multiple communication channels were used between management, caregivers and families”, including emails, factsheets, and signage. Most nursing homes had a visit from their attending physician two times weekly, though one institution specified that these visits were sometimes virtual. All interviewees reported that their attending physicians did not participate in either formal or informal information sessions.

**Figure 3:**
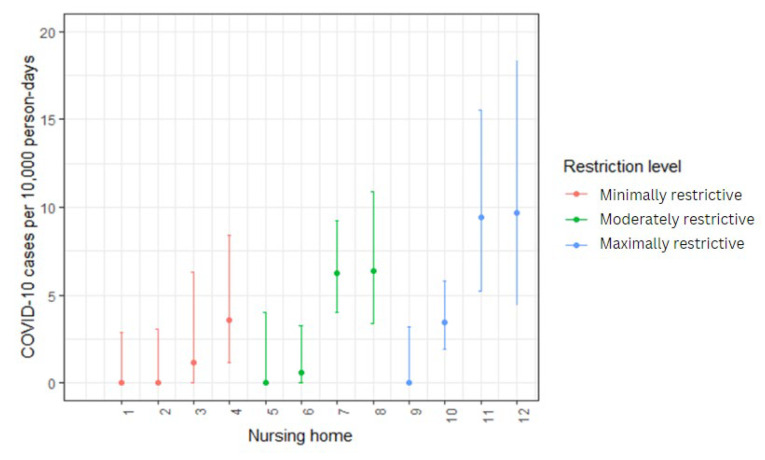
Incident rate ratios with 95% confidence intervals grouped by maximally, moderately, and minimally restrictive non-pharmaceutical interventions across 12 nursing homes in Geneva. The extent of NPIs implemented (thematically categorized as maximally, moderately, or minimally restrictive) was not shown to have a significant association on the cumulative incidence of COVID-19 cases among residents.

Of the 12 nursing homes interviewed, we observed an equivalent, one-third, distribution of maximally restrictive, moderately restrictive, and minimally restrictive NPIs. To assess if these themes are associated with more or less infection, we compared the three levels of COVID-19-related measures taken in these nursing homes, and the cumulative incidence of PCR-confirmed COVID-19 cases in their resident population (Figure 3). The incidence rate ratios (IRRs) for moderately and maximally restrictive measures were estimated to be 3.55 and 3.90, respectively, when compared to minimally restrictive NPIs as a reference (Table 1). These associations were not statistically significant.

## 4. Discussion

Semi-structured interviews with representation from one-fifth of nursing homes in Geneva revealed that most nursing homes mandated NPIs for their staff and residents during the first wave of COVID-19. Canton-specific recommendations for nursing homes largely included limited contact with visitors, social distancing where possible, increased hand hygiene, wearing facemasks in the presence of residents, coughing into tissue paper or one’s elbow, or restricting access to common spaces. Nursing homes in our study showed high variability in which NPIs, and to what extent, they implemented. There was an equal distribution of maximally, moderately, and minimally restrictive NPIs for COVID-19 for workers and residents between March and May 2020. This variation also appeared to persist over the three-month period, suggesting a temporal consistency, showing that nursing homes tended to maintain their chosen approach through the first wave of the pandemic. The authors infer that each Director’s perspective on balancing freedom and safety likely influenced the consistency and stringency of NPI implementation. To our knowledge, this is among the few studies that qualitatively describes COVID-19 transmission patterns in nursing homes as a function of NPIs. A key strength of our study is that all nursing homes were located within the same canton, and subject to the comparable health policies, financing, and socio-economic standards.

While some nursing homes implemented measures like visit restrictions and limitations on social activities to curb the spread of SARS-CoV-2, there is still limited evidence regarding their effectiveness. We were unable to establish a concrete link between how restrictive NPIs in nursing homes were and COVID-19 positivity in residents and/or staff. This is not surprising given that the positive association between staff seroprevalence and COVID-19 cases in residents also had large variability, as did staff seroprevalence between nursing homes [2,3]. This suggests that regardless of the NPIs adopted by nursing homes, the rate of infection appears to be randomly affected and other determinants of infectious disease transmission are at play. A study conducted in Switzerland identified several potential protective factors, including routine symptom screening of healthcare workers, controlled visitations, availability of single rooms, and isolation of COVID-19 patients in individual rooms [8]. However, the reasons why some institutions experienced severe outbreaks while others were largely unaffected remain unclear.

In our study population, 41% of nursing homes did not have formal screening methods in place for staff. Some studies suggest that staff members were the primary source of infection for residents, rather than the other way around, emphasizing the need for targeted interventions among healthcare workers to mitigate COVID-19 risks in long-term care facilities. The practice of employing staff members working in multiple long-term care institutions in a part-time capacity can contribute to intra- and inter-facility spread of SARS-CoV-2 [9]. Therefore, screening healthcare workers for COVID-19, even if asymptomatic, appears to be an effective preventive strategy [10]. In a more stringent response, some staff members of nursing homes in France decided to voluntarily confine themselves with their residents to reduce the risk of entry of SARS-CoV-2 into the facility [11]. This retrospective cohort study later found that nursing homes where staff remained confined with residents had lower numbers of COVID-19 cases and mortality rates compared to those reported in a national population-based survey of nursing homes [11].

Strict visitor regulation may decrease resident mortality, but it may have adverse effects on resident well-being [12]. Furthermore, the extent to which differing levels of restrictions protect residents from infection remains unclear. A Dutch guideline was developed to cautiously open nursing homes for family visitation during the COVID-19 pandemic, and this study reported no new cases among residents provided there was strict adherence to infection prevention and control measures [13]. Restrictions on visits may have lasting impacts on patients, families, and healthcare services beyond the duration of the pandemic itself. The level of global evidence on longer-term effects from visiting restrictions is low and warrants further research. Even if the results of our research project do not allow us to understand which measures are most efficient in reducing transmission from healthcare staff to resident, it points to a larger public health discussion on maintaining a trade-off between infection control interventions that, at times, can limit positive social interactions and impact overall well-being. This approach reflects the complexity of decision-making in public health crises, and the need for nuanced and adaptive response strategies that rely on determinants other than morbidity or mortality. The supply of personal protective equipment amplifies the gravity of these decisions. The interviewees reported a limited supply of surgical masks, which staff managed by wearing one mask for each twelve-hour shift. This situation exemplifies the need for robust supply chain management and resource allocation strategies within healthcare institutions, especially during times of heightened demand.

Our analysis is limited to the first wave of COVID-19, between March and May 2020. The retrospective design of the ad hoc questionnaire is also subject to social desirability and recall biases; we cannot guarantee the authenticity of answers, especially given that the nursing home physicians interviewed are liable to sanctions for negligence under the Swiss Law on the Management of Institutions for the Elderly. While not all nursing homes had documented policies or institutional records, some did (*n* = 5), which helped support interviewee responses. Additionally, to mitigate recall bias, the interviews were primarily conducted with nursing home Directors and the physicians in charge, who could provide informed perspectives based on their shared experiences and institutional knowledge. The nursing homes interviewed were a convenience sample, meaning we cannot entirely rule out selection bias; these institutions were either the most accessible or the most willing to take part in this study. However, the main reason for non-participation was a lack of time rather than specific institutional characteristics. Additionally, the data available indicate that SARS-CoV-2 infections were evenly distributed among both participating and non-participating nursing homes, suggesting that the risk of significant bias in our findings is limited. We cannot definitively exclude the possibility that institutions experiencing more severe outbreaks may have concurrently implemented more stringent NPIs. Inter- and intra-variability in nursing home operations and directives for their staff and residents render it challenging to estimate the extent of the efficacy of NPIs. We were unable to establish a concrete link, should it exist, between how restrictive NPIs in nursing homes were and COVID-19 positivity in residents and/or staff. This is primarily due to study design and low statistical power. Cumulative incidence may also be underestimated, as only symptomatic residents were tested, leaving asymptomatic cases unaccounted for. Inconsistencies in how nursing homes collect and report health information for their staff and residents may also influence the general scope of these data. Of note, we do not report testing frequency across institutions. It is important to reiterate that our study is predominantly qualitative. We assessed qualitative data through in vivo coding to observe trends and offer a foundation for future research into the application of NPIs in the nursing home setting.

## 5. Conclusions

During the COVID-19 waves in the nursing homes included in our study, the trade-off decision focused on balancing the implementation of restrictive NPIs to protect public health, which could have unintended negative effects, against adopting fewer NPIs to maintain a sense of normalcy while potentially increasing the risk of infection. This suggests a recognition that there is no one-size-fits-all solution; decision-makers should continually assess the situation, considering the evolving circumstances and the best available data to find the most appropriate trade-off between health protection and the preservation of well-being.

## Figures and Tables

**Table 1:** Association between levels of COVID-19-related measures taken in nursing homes and cumulative incidence of PCR-confirmed COVID-19 cases among residents in each nursing home.

COVID-19 Measures	IRR * (95% CI)	*p*-Value
Minimally restrictive	Ref	
Moderately restrictive	3.55 (0.75–41.42)	0.212
Maximally restrictive	3.90 (0.82–45.54)	0.184

PCR = polymerase chain reaction, IRR = incidence rate ratio, CI = confidence interval, Ref = reference. * Estimated by quasi-Poisson log-linear regression.

## Data Availability

Study data that underlie the results reported in this article can be made available to the scientific community after deidentification of individual nursing homes and participants, and upon submission of a data request application to the investigator board via the corresponding author.

## Appendix A

*Semi-Structured Interview*
Nursing Home: Name and Position of Nursing Home Representative: Date of Completion:

Section 1: Screening Strategies 1. During the first wave of COVID-19, between 1 March 2020 and 1 June 2020, did you implement local screening strategies (e.g., temperature measurement, RT-PCR testing)? If yes, please specify what was implemented, - for staff? - for residents? - for visitors? 2. How did you manage employees with a positive test or close contact with a positive case? 3. How did you handle employees that presented symptoms consistent with COVID-19? 4. How did you manage residents with a COVID-19-positive test result? 5. Between 1 March 2020, and 1 June 2020, how did you manage residents that presented symptoms consistent with COVID-19? (e.g., isolation, hospitalization, protective equipment for caregivers)
Section 2: The Facility 1. Did you experience a shortage of personal protective equipment (PPE) during the first wave, between 1 March 2020 and 1 June 2020? If yes, please provide details: - What type of PPE was lacking (e.g., surgical masks, N95 masks, gowns, face shields, safety goggles, hand sanitizer, gloves)? - How long did the shortage last? - How did you manage this shortage? - What PPE was eventually provided, and when?
Section 3: COVID-19 Preventive Measures Implemented in the Nursing Home Between March 2020 and June 2020 1. Did you have an epidemic protection plan for the nursing home before this outbreak? If yes, did this plan serve for the COVID-19 pandemic? 2. Did you restrict access to certain common areas? 3. Were in-person activities suspended? If yes, which ones were suspended, and which continued? 4. Did you implement social distancing measures among residents? 5. How did you ensure hand hygiene for staff in your facility (e.g., hand sanitizer dispensers in common areas, posters, training)? 6. How did you ensure hand hygiene for residents in your facility (e.g., hand sanitizer dispensers in rooms, posters, informational sessions)? 7. Did you have a mask-wearing protocol between 1 March 2020 and 1 June 2020? If yes, when and how was it implemented (e.g., for employees, residents, visitors)? 8. Did you ban/restrict visits? If yes, when and how (e.g., restricted visiting hours, limited number of visitors, physical contact allowed)? 9. Did you receive instructions from your nursing home association and/or the Department of Health or the cantonal physician regarding preventive measures against COVID-19? If yes, were you able to follow them? 10. Did you take other measures to limit contact between residents and between caregiving staff and residents (e.g., assigning specific staff to specific residents, resident groups, or areas within the nursing home)? 11. Did employees receive training on COVID-19 prevention measures? If yes, when, in what format, and was it mandatory or optional? 12. Did physicians undergo training for COVID-19? If yes, when, in what format, and was it mandatory or optional? 13. Did residents have the opportunity to participate in information sessions about COVID-19 (hygiene measures and social distancing)? If yes, when, in what format, and was it mandatory or optional? 14. In your opinion, what were the strengths and weaknesses of your nursing home in managing the COVID-19 health crisis? 15. Are you a part of a nursing home association or umbrella organization?

## Appendix B

*Demographic Survey*
Nursing Home: Name and Position of Nursing Home Representative: Date of Completion:

Section 1: Nursing Home Staff 1. How many people were present on-site in your facility between 1 March 2020 and 1 June 2020 (including temporary staff)? Physicians: Registered Nurses: Nursing Assistants, Health and Community Care Assistants: Other Caregivers: Social Workers: Socio-hotel Staff (meals, maintenance, etc.): Volunteers: Activity Team: Chaplain: 2. What percentage of healthcare staff work part-time? 3. How many * caregivers worked in your nursing home between 1 March 2020 and 1 June 2020? March: April: May: * This includes all nurses, nursing assistants, and community care assistants, and other general caregivers. This will help us calculate the resident-to-caregiver ratio during this period. 4. What is the age distribution of all employees? 5. What is the gender distribution of all employees?
Section 2: Physicians 1. How many times per week did the physician associated with your facility and involved in institutional life visit the nursing home during the period from 1 March 2020 to 1 June 2020?
Section 3: Residents 2. What is the age distribution of residents? 3. What is the gender distribution of residents? 4. What was the number of beds and the occupancy rate per month between 1 March 2020 and 1 June 2020? March: April: May: 5. Did you prohibit visits from family/relatives during the period from 1 March 2020 to 1 June 2020? If yes, between which dates?
Section 4: The Facility 1. How many individual rooms are there in the nursing home? How many shared rooms are there in the nursing home?
